# Investigation of Surface Pre-Treatment Methods for Wafer-Level Cu-Cu Thermo-Compression Bonding

**DOI:** 10.3390/mi7120234

**Published:** 2016-12-15

**Authors:** Koki Tanaka, Wei-Shan Wang, Mario Baum, Joerg Froemel, Hideki Hirano, Shuji Tanaka, Maik Wiemer, Thomas Otto

**Affiliations:** 1 Department of Robotics, Tohoku University, 6-6-01, Aza Aoba, Aramaki Aoba-ku, Sendai 980-8579, Japan; koki-tanaka@mems.mech.tohoku.ac.jp (K.T.); tanaka@mems.mech.tohoku.ac.jp (S.T.); 2 Fraunhofer Institute for Electronic Nano Systems (ENAS), Technology Campus 3, Chemnitz 09126, Germany; wei-shan.wang@enas.fraunhofer.de (W.-S.W.); Mario.Baum@enas.fraunhofer.de (M.B.); thomas.otto@enas.fraunhofer.de (T.O.); 3 World Premier International Research Center Initiative-Advanced Institute for Materials Research, Tohoku University, 2-1-1 Katahira, Aoba-ku, Sendai 980-8577, Japan; joerg.froemel@mems.mech.tohoku.ac.jp; 4 Micro System Integration Center (µSIC), Tohoku University, 6-6-01, Aza Aoba, Aramaki Aoba-ku, Sendai 980-8579, Japan; hirano@mems.mech.tohoku.ac.jp; 5 Center for Microtechnologies (ZfM), Technische Universität Chemnitz, Reichenhainer Str. 70, Chemnitz 09126, Germany

**Keywords:** wafer bonding, thermo-compression bonding, pre-treatment, Cu-Cu bonding, 3D integration

## Abstract

To increase the yield of the wafer-level Cu-Cu thermo-compression bonding method, certain surface pre-treatment methods for Cu are studied which can be exposed to the atmosphere before bonding. To inhibit re-oxidation under atmospheric conditions, the reduced pure Cu surface is treated by H_2_/Ar plasma, NH_3_ plasma and thiol solution, respectively, and is covered by Cu hydride, Cu nitride and a self-assembled monolayer (SAM) accordingly. A pair of the treated wafers is then bonded by the thermo-compression bonding method, and evaluated by the tensile test. Results show that the bond strengths of the wafers treated by NH_3_ plasma and SAM are not sufficient due to the remaining surface protection layers such as Cu nitride and SAMs resulting from the pre-treatment. In contrast, the H_2_/Ar plasma–treated wafer showed the same strength as the one with formic acid vapor treatment, even when exposed to the atmosphere for 30 min. In the thermal desorption spectroscopy (TDS) measurement of the H_2_/Ar plasma–treated Cu sample, the total number of the detected H_2_ was 3.1 times more than the citric acid–treated one. Results of the TDS measurement indicate that the modified Cu surface is terminated by chemisorbed hydrogen atoms, which leads to high bonding strength.

## 1. Introduction

Wafer-level bonding has been one of the key technologies for 3D integration. Among various wafer bonding technologies, Cu-Cu thermo-compression bonding has been an attractive choice in terms of its compatibility for microelectronics metallization and its lower cost, for example as compared to surface-activated bonding and Au-Au bonding from the bonding system and material points of view, respectively.

Basic research for thermo-compression bonding was conducted with materials other than Cu in aeronautics and space applications, e.g., titanium alloys. Ashby, Derby, and Takahashi showed principal investigations with a typical process flow for diffusion welding: defectless flow, dislocation glide, diffusional creep, and dislocation creep. Takahashi built a model to simulate the void closure in between the bond interface during bonding [1,2,3]. The bonding mechanism of thermo-compression bonding is achieved through atomic contact, diffusion and grain growth as well as temperature impact and pressure. Frömel investigated different intermediate materials such as Au, Cu and Al for thermo-compression bonding [4].

The surface quality with respect to flatness, homogeneity, and cleanliness plays an important role in thermo-compression bonding. Chen reported on wafer-level Cu-Cu thermo-compression bonding with the removal of Cu native oxide by using HCl solution as a pre-treatment [5,6]. A thin native oxide may form again between the HCl pre-treatment and the bonding process. At least a 350 °C bonding temperature with post-bond annealing is necessary to obtain good bonding strength [7]. Baum adopted two pre-treatment processes before Cu-Cu bonding to achieve hermetic sealing [8]. Rebhan used different pre-treatments to decrease the bonding temperature significantly [9]. Shigetou and Yang showed that low-temperature Cu-Cu bonding below 250 °C can be achieved by using a special in situ pre-treatment setup such as Ar fast atom beam bombardment and formic acid vapor treatment [10,11]. With the in situ pre-treatment methods, the removal of Cu native oxide and Cu-Cu bonding were sequentially performed in the same vacuum ambient. Thus, a pair of Cu surfaces was contacted without native oxide. However, such a special in situ setup bonding system is complicated and expensive. Additionally, substrate damage, such as physical damage by Ar atom bombardment and metal corrosion by formic acid, is also an issue.

Tan and Lim achieved low-temperature Cu-Cu bonding below 250 °C using the usual bonding system [12,13]. The pure Cu surface was covered by thiol-based self-assembled monolayer (SAM) and not re-oxidized even in the atmosphere. The bonding was performed after the removal of SAM film by heating in the vacuum. Such a surface protection method is quite attractive because of the simple bonding system and flexible fabrication process. In this paper, several attractive pre-treatment methods which can inhibit the re-oxidation of Cu in the atmosphere are performed and investigated.

## 2. Experimental Design and Bonding Process

### 2.1. Sample Wafer Fabrication

**Figure 1:**
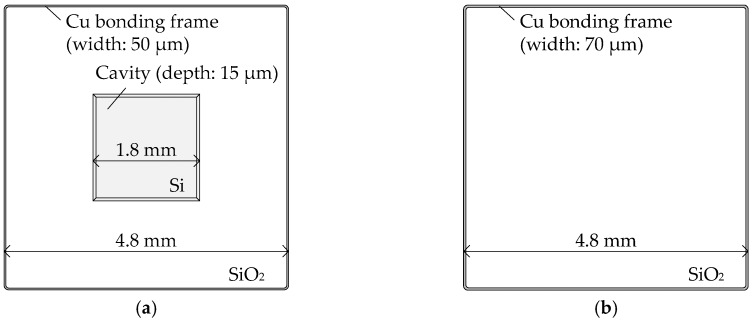
Overview of the Cu metallization patterns. (**a**) Bottom side wafer; (**b**) top side wafer.

Cu-Cu bonding was carried out by using a bottom-side wafer and a top-side wafer which had Cu bonding frame structures. Cu metallization patterns of a bottom-side wafer and a top-side wafer are shown in Figure 1. The bottom-side bonding frames (line width: 50 µm, 4.8 mm square) and the top-side bonding frames (line width: 70 µm, 4.8 mm square) were fabricated on a pair of 6 in wafers. Each bottom-side bonding pattern has a cavity (depth: 15 µm, 1.8 mm square) to avoid Si-Si direct contact with the top-side wafer.

**Figure 2:**
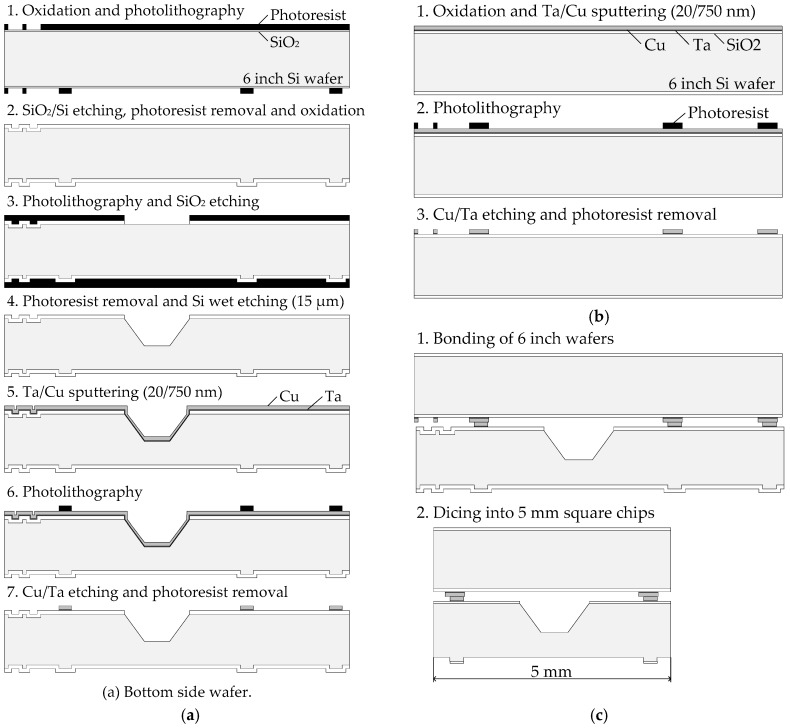
Fabrication process of the sample wafers. (**a**) Bottom side wafer; (**b**) top side wafer; (**c**) bonding and dicing process.

The fabrication processes of the sample wafers are shown in Figure 2. Ta and Cu were sputtered on a Si wafer. Ta was deposited as a diffusion barrier layer. The bonded wafers were diced into 5 mm square chips and tested by tensile tests.

### 2.2. Pre-Treatment and Bonding Process

A pair of the wafers was bonded by a bonding system (EVG 540, EVG, St. Florian, Austria) after a pre-treatment process. The conditions of the pre-treatment methods are shown in Table 1. Two plasma pre-treatment methods and SAM pre-treatment methods are based on the inhibition of Cu re-oxidation in the atmosphere. Formic acid vapor and citric acid pre-treatment are the methods for oxide removal and were carried out to compare with other methods.

The formic acid vapor pre-treatment was the only method in this paper which can be directly performed in the bonder. A pair of the wafers was pre-treated and bonded in the vacuum ambient consecutively, which indicates that bonding should be performed without the Cu native oxide. A pair of the wafers was introduced into the chamber, and heated up to 30 °C, 150 °C and 300 °C, consecutively. Formic acid vapor was introduced for 10 min at each temperature.

On the other hand, plasma-, SAM- and citric acid-treated wafers were exposed to the atmosphere for 30 min before bonding, where relative humidity and room temperature were controlled at approximately 40% and 25 °C, respectively. The citric acid solution pre-treatment was carried out as a single wet process, which cannot inhibit Cu re-oxidation in the atmosphere. A pair of the wafers was immersed into 1 wt % citric acid solution in deionized (DI) water for 1 min, and rinsed by DI water.

The H_2_/Ar and NH_3_ plasma pre-treatments were expected to be able to reduce Cu native oxide and form surface protection layers at the same time. Both of the plasmas can reduce Cu native oxide effectively by generating hydrogen radicals. In addition, a Cu hydride–like layer and Cu nitride layer (Cu_3_N) should be formed by the H_2_/Ar and NH_3_ plasma, respectively [14,15]. Thus, Ar gas is not necessary for this process, but it was introduced to stabilize plasma. These plasma pre-treatments were carried out by a plasma processing system (Applied Precision 5000, Applied Materials, Inc., Santa Clara, CA, USA).

The SAM pre-treatments were performed after the removal of Cu native oxide by the citric acid treatment, because the SAM process cannot remove the Cu native oxide. After the removal of the oxide, a pair of wafers was rinsed by DI water, and immediately immersed into the SAM solution. After storing for 2 h, the wafers were rinsed by 2-propanol and DI water. We tried to remove the SAM film by heating in the bonding chamber.

The bonding conditions are shown in Table 2, where only the bonding temperature is varied. A pair of the wafers was introduced into the bonding chamber after alignment in the atmosphere. In the bonding chamber, the wafers were first heated, and then contacted. In the case of SAM pre-treated wafers, additional heating at 200 °C for 10 min was performed to remove the SAM film before bonding. The distance of a pair of aligned wafers was 50 µm, where the formic acid vapor pre-treatments and removal of SAM film were performed.

## 3. Bonding Results and Discussion

### 3.1. Tensile Test

**Figure 3:**
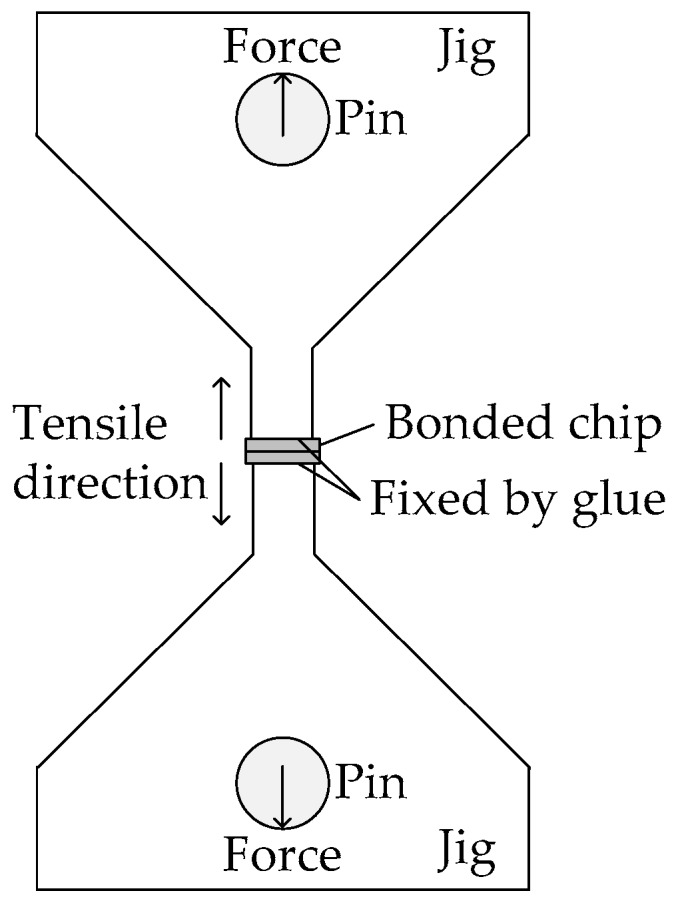
Schematic diagram of the tensile test setup.

The strength of the bonded chips was evaluated by the tensile test. A schematic of the tensile test setup is shown in Figure 3. A bonded chip was fixed to jigs by glue (ethyl cyanoacrylate) and pulled in a vertical direction. Tensile tests were carried out by TIRA test 2805. It should be noted that the measured tensile strength using this setup is not the actual tensile strength of the Cu-Cu bonded interface. Each Si substrate was bended during the tensile test, because the bonding frames were placed on the edge of the chips. Thus, the shear stress on the bonding frames will be increased with the deformation of the Si substrates. In addition, applied stress should be concentrated on the corners of the bonding frames due to the deformation of the Si substrates.

**Figure 4:**
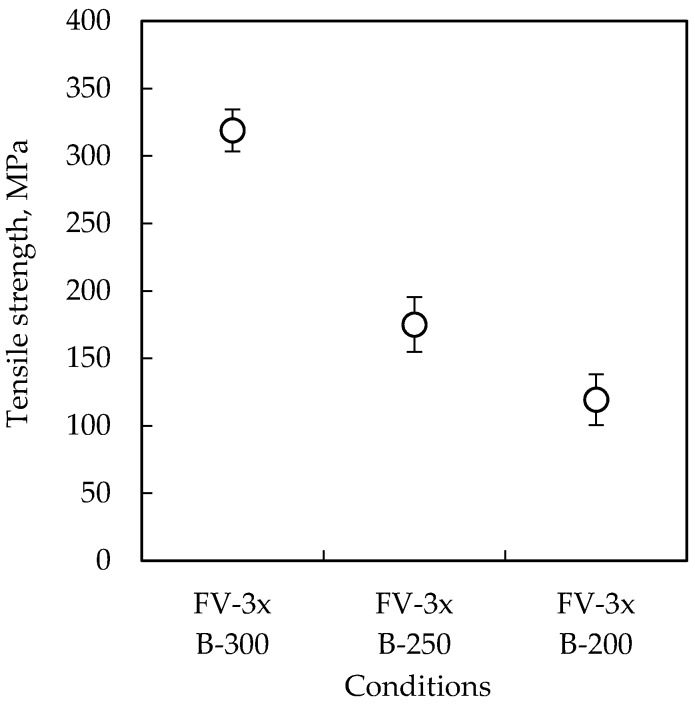
Tensile strengths of the formic acid vapor pre-treated chips at different bonding temperatures. Nine chips were tested in each condition.

The tensile strengths of the formic acid vapor pre-treated chips at different bonding temperatures are shown in Figure 4. The chips bonded at 300 °C show a bonding strength of 320 MPa, which is at the same level of the bulk Cu-Cu diffusion bonding strength, but much higher than the reported wafer-level Cu-Cu bonding strength in the range of 20–40 MPa [6,16]. It is considered that the actual bonding strength should be lower than the measured strength for the reasons mentioned above. Anyway, the chips bonded at 200 °C and 250 °C were fractured from the Cu-Cu interface, but the chips bonded at 300 °C were fractured from the adhesion layer or the Si substrate. Thus, at least a 300 °C bonding temperature is suggested even if the Cu is not covered by oxide.

**Figure 5:**
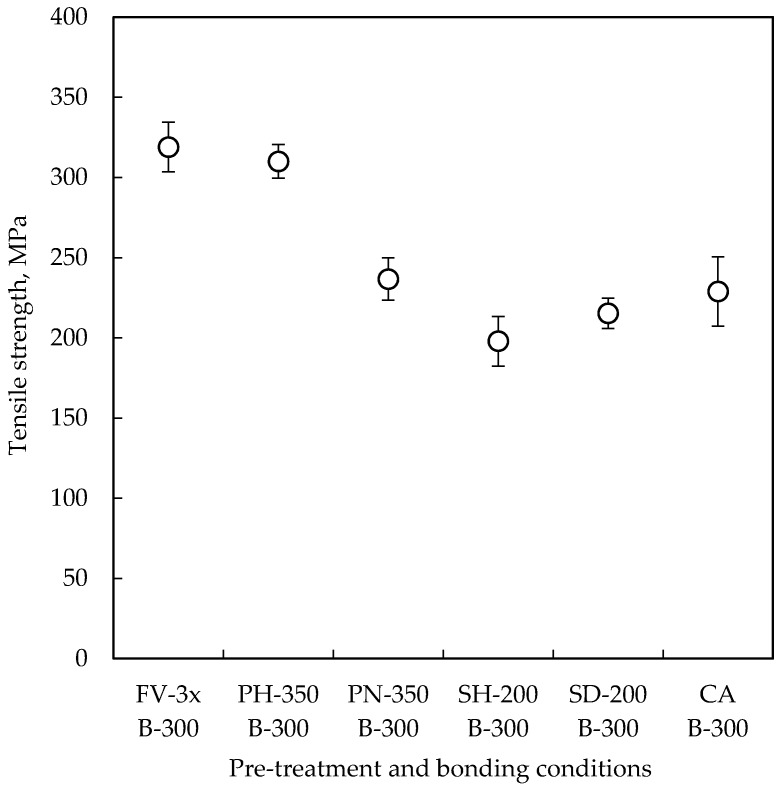
Tensile strengths of the chips bonded at 300 °C with different pre-treatment methods. Nine chips were tested in each condition.

Most of the chips bonded at 200 °C and 250 °C with other pre-treatment methods were separated during the dicing process. On the other hand, all of the chips bonded at 300 °C were not separated, and they could only be measured, as shown in Figure 5. The H_2_/Ar plasma pre-treated chips show the same level of tensile strength as the formic acid vapor pre-treated chips, even when exposed to the atmosphere for 30 min. On the other hand, the NH_3_ plasma, hexanethiol SAM and decanethiol SAM pre-treated chips show comparable bond strengths as the citric acid pre-treated chips.

**Figure 6:**
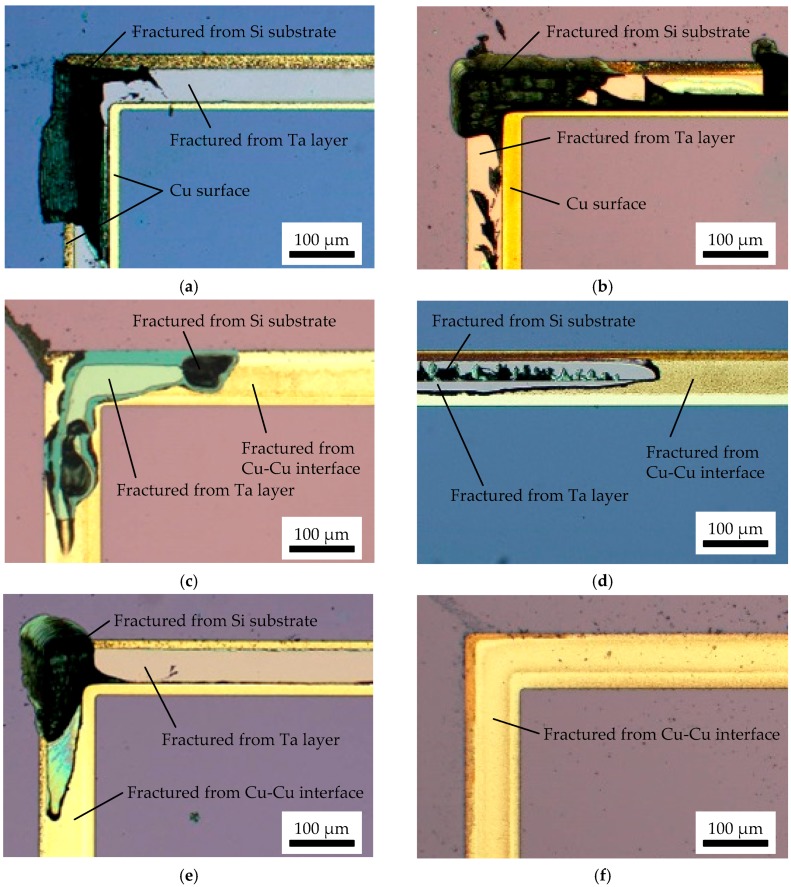
Microscope images on fractured interface of the tensile-tested chips. The chips were bonded at 300 °C. (**a**) Formic acid vapor pre-treated chip; (**b**) H_2_/Ar plasma pre-treated chip; (**c**) NH_3_ plasma pre-treated chip; (**d**) hexanethiol SAM pre-treated chip; (**e**) decanethiol SAM pre-treated chip; (**f**) citric acid solution pre-treated chip.

The fractured interfaces of the tested chips are observed by microscope, as shown in Figure 6. In the case of formic acid vapor and H_2_/Ar plasma pre-treatments, the chips were all fractured from the Si substrate or Ta layer, which indicates that the Cu-Cu interfaces are strongly bonded. The reason that the H_2_/Ar plasma pre-treated wafers can be bonded even when exposed to the atmosphere is because of the hydride-like layer that is formed on the reduced pure Cu surface. Baklanov observed the H_2_ plasma–treated Cu surface using in situ ellipsometry, and found that the ellipsometric characteristics of pure Cu surface were changed by H_2_ plasma treatment [14]. They supposed that a hydrogen-rich and nontransparent Cu hydride–like layer was formed on the pure Cu surface layer. It is also analyzed in the following thermal desorption spectroscopy (TDS) measurements.

In contrast, the citric acid pre-treated chips are all fractured from the Cu-Cu interface, which implies that the Cu molecules on each surface are not diffused due to the Cu native oxide layer. On the other hand, the NH_3_ plasma and SAM pre-treated chips show different fracture conditions. They are usually fractured from the Cu-Cu interface, but some are fractured from the Si substrate or Ta layer. This indicates that there are strongly bonded areas and non-bonded areas. It is considered that the surface protection layer such as Cu nitride and SAM could inhibit re-oxidation, but it is not completely removed before bonding. In fact, around a 350 °C temperature is required to decompose Cu_3_N to Cu and N_2_ [17]. On the other hand, it is reported that 200 °C heating for 10 min is enough to desorb SAM film [12,18]. However, the distance of a pair of the Cu surfaces is 50 µm in this experiment. The removed thiol compounds could be re-adsorbed on the opposite side of the Cu surface due to this small distance. To eliminate the influence of the small gap between the wafers, heating for SAM removal must be done before the alignment in the vacuum ambient, which makes the bonding system more complicated.

### 3.2. Thermal Desorption Spectroscopy (TDS) Measurements

**Figure 7:**
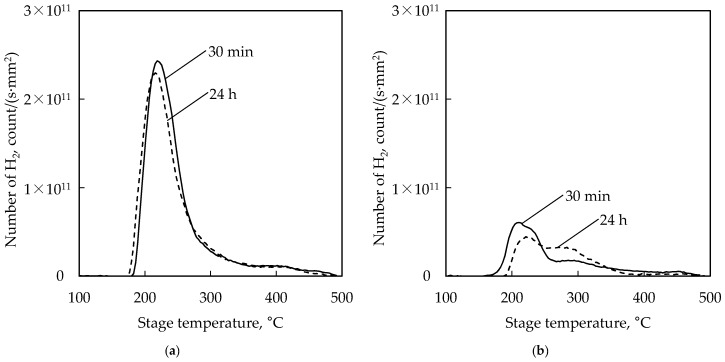
Change of the TDS spectrum with time in the atmosphere. After the treatments, Cu film was exposed to the atmosphere for 30 min and 24 h. The spectra show the H_2_ desorption rate along with the increasing temperature. The temperature elevation rate was 60 °C/min. (**a**) H_2_ desorption spectrum of H_2_/Ar plasma treated Cu; (**b**) H_2_ desorption spectrum of citric acid treated Cu.

To clarify the reason for strongly bonded H_2_/Ar plasma pre-treated chips, TDS measurements were carried out by TDS equipment (TDS1200, ESCO, Ltd., Tokyo, Japan) which can estimate the desorbed number of molecules [19,20]. Ta (20 nm) and Cu (750 nm) sputtered Si substrates (width: 10 × 10 mm, thickness: 400 µm) were prepared as sample substrates, and treated by the H_2_/Ar plasma and the citric acid solution. The TDS spectra of the sample substrates are shown in Figure 7.

The TDS spectra show that approximately 3.1 times greater H_2_ molecules were desorbed from the H_2_/Ar plasma–treated Cu film as compared to the citric acid–treated one. It is considered that these H_2_ molecules were derived from chemisorbed hydrogen atoms on the Cu films, because the depth of the potential energy well is quite shallow (20 meV) in the case of H_2_ physical adsorption on Cu [21]. Usually, hydrogen molecules were easily dissociated on metal surfaces by the catalytic effect. In the case of Cu, molecular hydrogen cannot be dissociated and chemisorbed without an extra portion of energy. In contrast, atomic hydrogen is immediately chemisorbed on the Cu surface without any barriers of activation energy [22]. Therefore, the desorbed hydrogen molecules from the H_2_/Ar plasma–treated Cu film are considered to be derived from hydrogen radicals, which are generated by the H_2_/Ar plasma. In addition, it supposes that the Cu hydride–like layer which is mentioned by Baklanov was formed as hydrogen atoms chemisorbed on the Cu layer [14]. It is suggested that the oxidation of the treated Cu surface was suppressed by the formed Cu hydride–like layer, because some of the dangling bonds on the pure Cu surface were terminated by hydrogen atoms.

As shown in Figure 7a, the H_2_ desorption rate of the H_2_/Ar plasma–treated Cu film was not significantly changed even when exposed for 24 h. A small number decreasing of H_2_ desorption is considered to be derived from desorption due to the growth of Cu oxide, which forms a more stable state. Thus, this chemisorption state of atomic hydrogen is relatively stable, and can be kept at least 24 h even in the atmosphere, because the well depth value of a hydrogen atom on Cu is sufficiently deep, e.g., it is calculated by the first principle calculation as approximately 2.5 eV on a hollow site of the Cu(111) surface [23,24]. It is suggested that high-strength Cu-Cu bonding is possible even when exposed for 24 h.

## 4. Conclusions

Wafer-level Cu-Cu bonding using various pre-treatment methods is demonstrated. A pair of H_2_/Ar plasma pre-treated wafers can be bonded even when exposed to the atmosphere for 30 min. In addition, the bonded chips showed a bond strength of 310 MPa, which is at the same level of the formic acid vapor pre-treated chips. On the other hand, the tensile strengths of the NH_3_ plasma and SAM pre-treated chips are comparable to the citric acid pre-treated chips. The lower bond strength might be due to the remaining Cu_3_N layer and thiol compounds on the Cu surface.

The reason why the H_2_/Ar plasma pre-treated wafers could be bonded strongly as suggested by the TDS measurements is that oxidation of the treated Cu surface was suppressed by chemisorbed hydrogen atoms. In the case of the H_2_/Ar plasma–treated Cu film, the total desorption number of H_2_ was approximately 3.1 times greater than the citric acid–treated one.

Results show that the Cu-Cu bonding process using H_2_/Ar pre-treatment can be carried out in a typical bonding system even after 30 min of exposure to the atmosphere, which is enough to transfer the wafers from the plasma equipment to a bonder. In addition, hydrogen radicals do not attack commonly used materials such as metals, ceramics and Si-based low-*k* dielectrics, except for some organic materials such as organic low-*k* dielectrics [14]. Therefore, H_2_/Ar or H_2_ plasma pre-treatment is very promising from the view point of simple bonding systems, flexible processes and mild pre-treatment conditions.

## Figures and Tables

**Table 1:** Pre-treatment conditions of Cu-Cu thermo-compression bonding.

Pre-Treatment	Code	Temperature	Time	Remarks
Formic acid vapor	FV-3x	300 °C	3 × 10 min	Three-step heating up to 300 °C, each heating step takes 10 min. Pressure of formic acid vapor was 100 kPa. Performed in the bonding chamber and bonded without Cu oxide layer to compare others.
H_2_/Ar plasma	PH-350	350 °C	30 s	H_2_/Ar flow rate, RF power and pressure were 300/140 sccm, 100 W and 130 Pa, respectively. Oxide can be removed by H radical and Ar ion. H atom chemisorption on Cu surface is expected.
NH_3_ plasma	PN-350	350 °C	60 s	NH_3_ flow rate, RF power and pressure were 220 sccm, 100 W and 130 Pa, respectively. Oxide can be removed by radicals. Cu_3_N is expected to be formed on Cu surface.
Hexanethiol SAM	SH-200	Room temperature	2 h	1-Hexanethiol (95%) was dissolved in 2-propanol at 1 mM of concentration. Native oxide was removed by citric acid. SAM film was tried to be desorbed by heating at 200 °C for 10 min before bonding.
Decanethiol SAM	SD-200	Room temperature	2 h	1-Decanethiol (95%) was dissolved in 2-propanol at 1 mM of concentration. Native oxide was removed by citric acid. SAM film was tried to be desorbed by heating at 200 °C for 10 min before bonding.
Citric acid solution	CA	Room temperature	1 min	1 wt % citric acid solution in DI water. A pair of the wafers was immersed into the citric acid solution, and bonded with re-oxidized Cu oxide layer to compare with others.

**Table 2:** Bonding conditions of Cu-Cu thermo-compression bonding.

Code	Temperature	Force	Duration
B-300	300 °C	30 kN	60 min
B-250	250 °C	30 kN	60 min
B-200	200 °C	30 kN	60 min
